# Supplementary material for: Halogen Bonding Involving Isomeric Isocyanide/Nitrile Groups
Source: Int J Mol Sci. 2023 Aug 28;24(17):13324. doi: 10.3390/ijms241713324 (PMC10487382; doi:10.3390/ijms241713324)
Supplement: Supplementary file 1 [file ijms-24-13324-s001.zip › ESI_SUBM.pdf]

Electronic Supporting Materials

Halogen Bonding Involving Isomeric Isocyanide/Nitrile Groups

Andrey S. Smirnov [1], Eugene A. Katlenok [1], Alexander S. Mikherdov [1], Mariya A. Kryukova [1], Nadezhda A. Bokach [1], Vadim Yu. Kukushkin [1,2]*

[1] Institute of Chemistry, Saint Petersburg State University, Universitetskaya Nab. 7/9, 199034 Saint Petersburg, Russian Federation

[2] South Ural State University, 76, Lenin Av., 454080 Chelyabinsk, Russian Federation

*Correspondence: v.kukushkin@spbu.ru

TABLE OF CONTENTS

CRYSTAL DATA	3
COMPUTATIONAL DETAILS	4
FTIR-ATR, PDXRD, AND TG DATA	5
CARTESIAN COORDINATES FOR THE STUDIED COCRYSTALS	14

Crystal data

Table S1 Crystal data for **1**·1,3,5-FIB, **2**·2(1,3,5-FIB), and **3**·2(1,3,5-FIB).

Identification code	**1**·1,3,5-FIB	**2**·2(1,3,5-FIB)	**3**·2(1,3,5-FIB)
Empirical formula	$C_{18}H_{12}F_3I_3N_2$	$C_{10}H_3F_3I_3N$	$C_{10}H_2F_3I_3N$
Formula weight	694.00	574.83	573.83
Temperature/K	100.15	100(2)	100.0(3)
Crystal system	monoclinic	monoclinic	monoclinic
Space group	$P2_1/c$	$P2_1/n$	$P2_1/n$
a/Å	13.2428(2)	7.9630(3)	7.8302(2)
b/Å	17.1531(3)	13.0858(4)	13.2296(3)
c/Å	9.4351(2)	12.9186(4)	12.8690(3)
α/°	90	90	90
β/°	107.514(2)	101.268(3)	100.464(2)
γ/°	90	90	90
Volume/Å^3	2043.88(7)	1320.20(8)	1310.93(5)
Z	4	4	4
ρ_{calc}g/cm^3	2.255	2.892	2.907
μ/mm^{-1}	36.316	55.952	56.347
F(000)	1280.0	1024.0	1020.0
Crystal size/mm^3	0.12 × 0.07 × 0.03	0.03 × 0.01 × 0.01	0.15 × 0.14 × 0.1
Radiation	CuKα (λ = 1.54184)	Cu Kα (λ = 1.54184)	Cu Kα (λ = 1.54184)
2Θ range for data collection/°	7 to 138.336	9.716 to 138.182	9.672 to 144.962
Index ranges	$-15 \le h \le 16, -19 \le k \le 20, -11 \le l \le 10$	$-9 \le h \le 8, -15 \le k \le 15, -15 \le l \le 15$	$-9 \le h \le 7, -16 \le k \le 16, -15 \le l \le 15$
Reflections collected	10729	8038	17886
Independent reflections	3818 [R_{int} = 0.0458, R_{sigma} = 0.0410]	2453 [R_{int} = 0.0468, R_{sigma} = 0.0419]	2600 [R_{int} = 0.0994, R_{sigma} = 0.0533]
Data/restraints/parameters	3818/0/239	2453/0/154	2600/0/154
Goodness-of-fit on F^2	1.052	1.058	1.067
Final R indexes [I>=2σ (I)]	R_1 = 0.0369, wR_2 = 0.0950	R_1 = 0.0290, wR_2 = 0.0723	R_1 = 0.0353, wR_2 = 0.0914
Final R indexes [all data]	R_1 = 0.0383, wR_2 = 0.0965	R_1 = 0.0330, wR_2 = 0.0742	R_1 = 0.0386, wR_2 = 0.0940
Largest diff. peak/hole / e Å^{-3}	1.03/-2.02	1.03/-0.90	1.35/-1.30
CCDC number	2281032	2280076	2280077

Table S2. Selected bond lengths and angles for **1**·1,3,5-FIB, **2**·2(1,3,5-FIB), and **3**·2(1,3,5-FIB).

**1**·1,3,5-FIB	d(N≡C), Å	1.152
	d(C–I), Å	2.088
	d(C···I), Å	3.035
	∠(C···I–C),°	173.38
**2**·2(1,3,5-FIB)	d(N≡C), Å	1.157
	d(C–I), Å	2.097
	d(C···I), Å	2.957
	∠(C···I–C),°	172.0
**3**·1,3,5-FIB	d(C≡N), Å	1.145(8)
	d(C–I), Å	2.100(5)
	d(C···I), Å	2.935(5)
	∠(C···I–N),°	173.79(19)

Computational details

Table S3. Main geometric parameters of the optimized structures.

Clusters		
[**1**·1,3,5-FIB]	d(N≡C), Å	1.1645
**1**	d(N≡C), Å	1.1678
[**1**·1,3,5-FIB]	d(C–I), Å	2.0931
1,3,5-FIB	d(C–I), Å	2.0778
[**1**·1,3,5-FIB]	d(I···C), Å	3.0678
[**1**·1,3,5-FIB]	∠(I···C),°	178.70
[**2**·1,3,5-FIB]	d(N≡C), Å	1.1647
**2**	d(N≡C), Å	1.1684
[**2**·1,3,5-FIB]	d(C–I), Å	2.0913
[**2**·1,3,5-FIB]	d(I···C), Å	3.0382
[**2**·1,3,5-FIB]	∠(I···C),°	178.08
[**3**·1,3,5-FIB]	d(C≡N), Å	1.1498
**3**	d(C≡N), Å	1.1511
[**3**·1,3,5-FIB]	d(C–I), Å	2.0840
[**3**·1,3,5-FIB]	d(I···N), Å	3.0433
[**3**·1,3,5-FIB]	∠(I···N),°	178.32

FTIR-ATR, PDXRD, and TG data

Table S4. Selected bands in the FTIR-ATR spectra.

**Compound/Cocrystal**	**ν(C≡N) or ν(N≡C), cm^{-1}; [Δν]**	
	Experimental value	Calculated value
**1**	2112	2218 [20]
**1**·(1,3,5-FIB)	2130 [18]	2238, 2216* [22]*
**2**	2130	2221 [23]
**2**·2(1,3,5-FIB)	2143 [13]	2244, 2220* [24]*
**3**	2232	2364 [7]
**3**·2(1,3,5-FIB)	2233 [1]	2371, 2364* [7]*

Δν = ν(cocrystals) – ν(**1**–**3**)

* CN stretching frequencies group of that are not involved in the HaB.

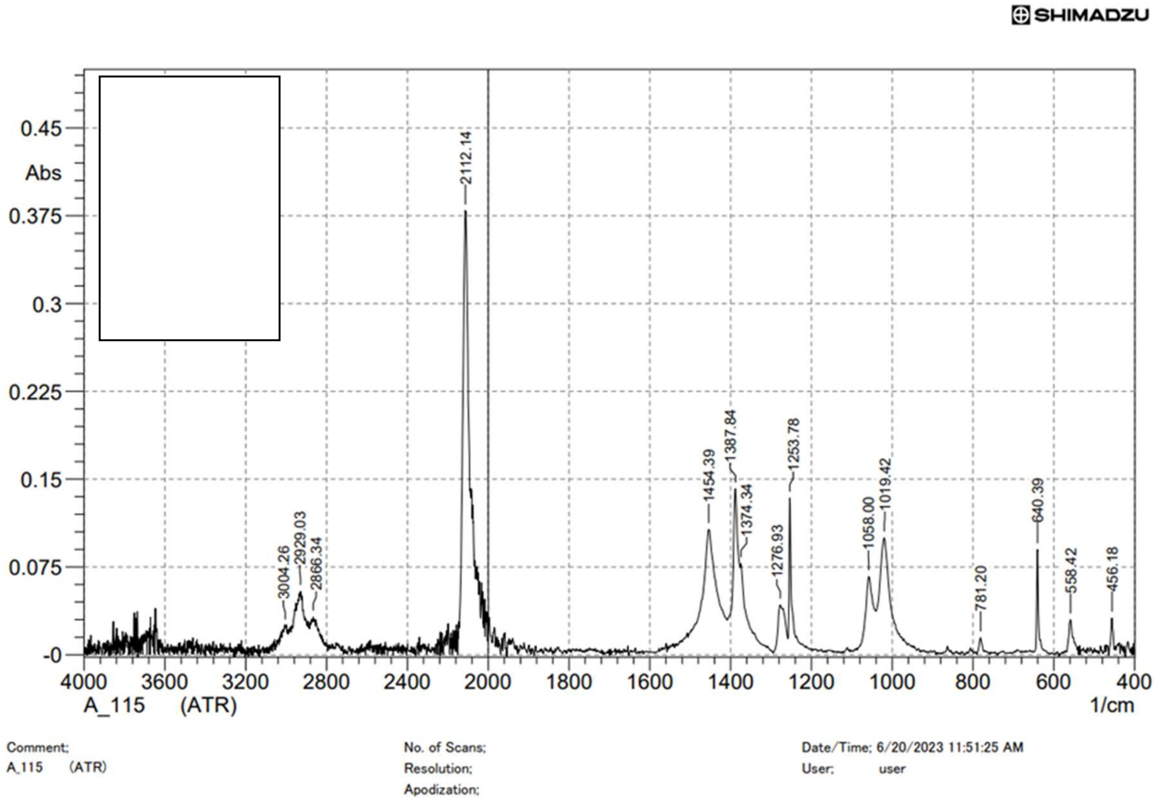

Figure S1. FTIR-ATR spectrum of **1**.

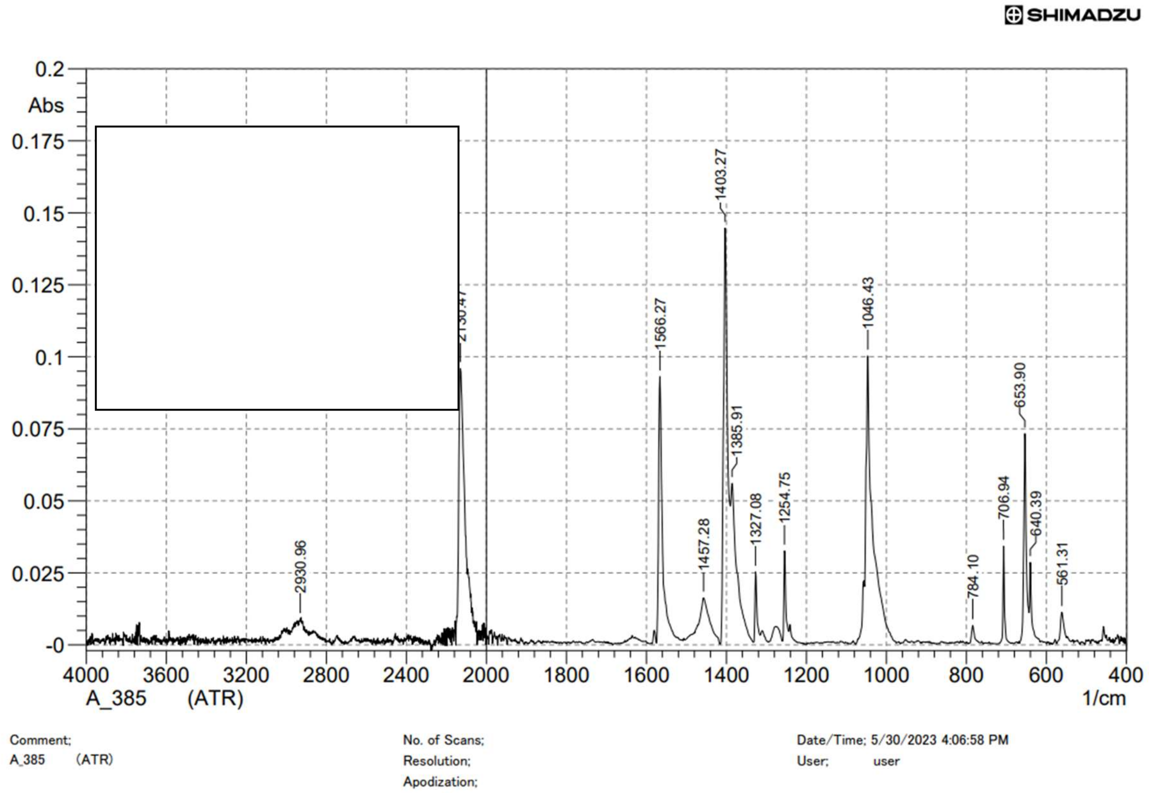

Figure S2. FTIR-ATR spectrum of **1**·(1,3,5-FIB).

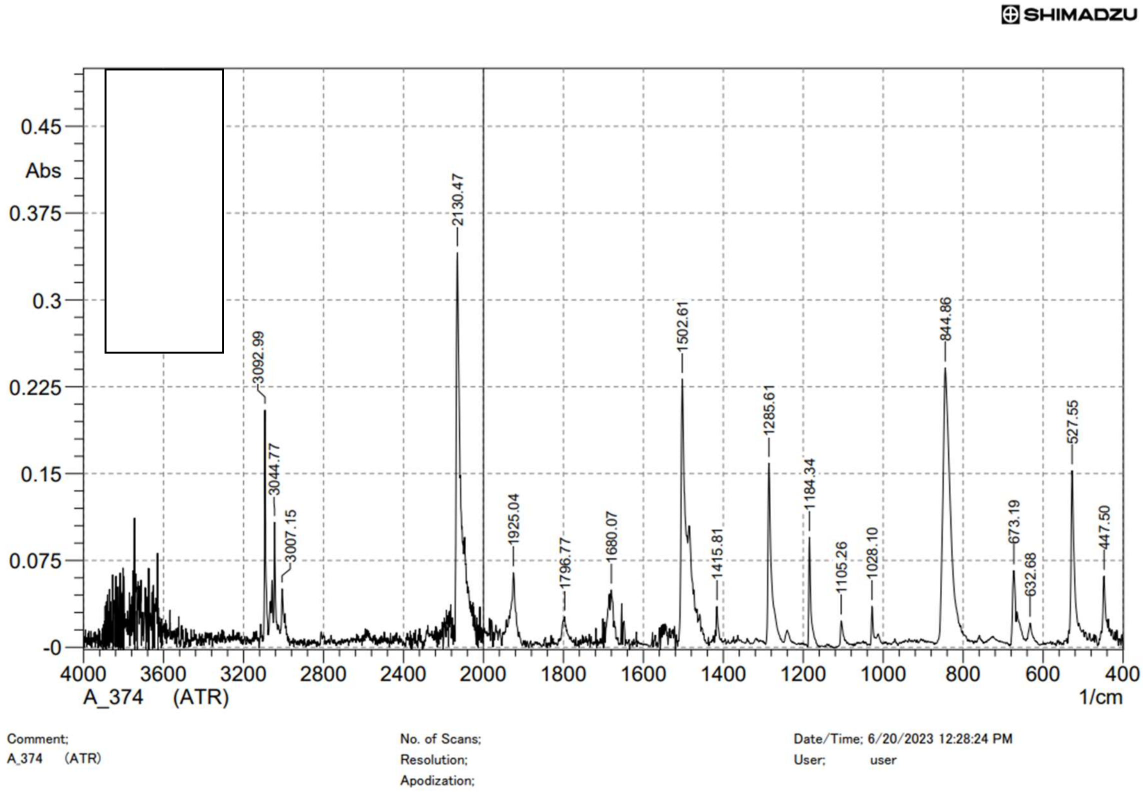

Figure S3. FTIR-ATR spectrum of **2**.

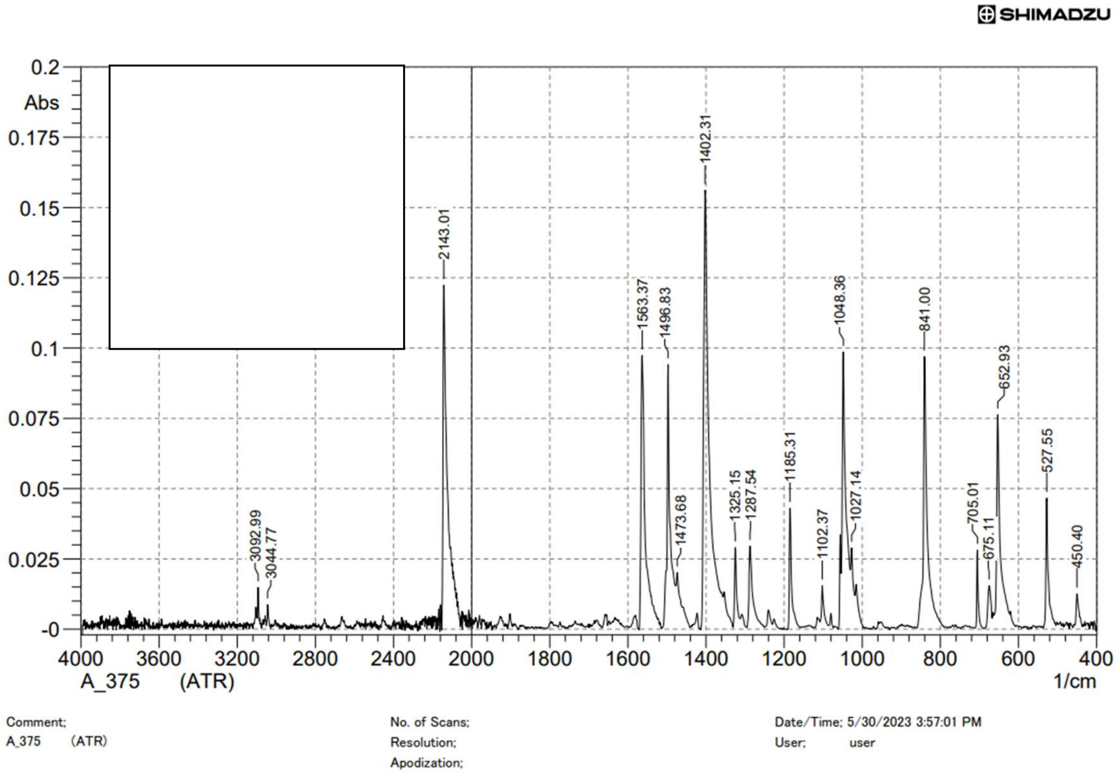

Figure S4. FTIR-ATR spectrum of **2**·2(1,3,5-FIB).

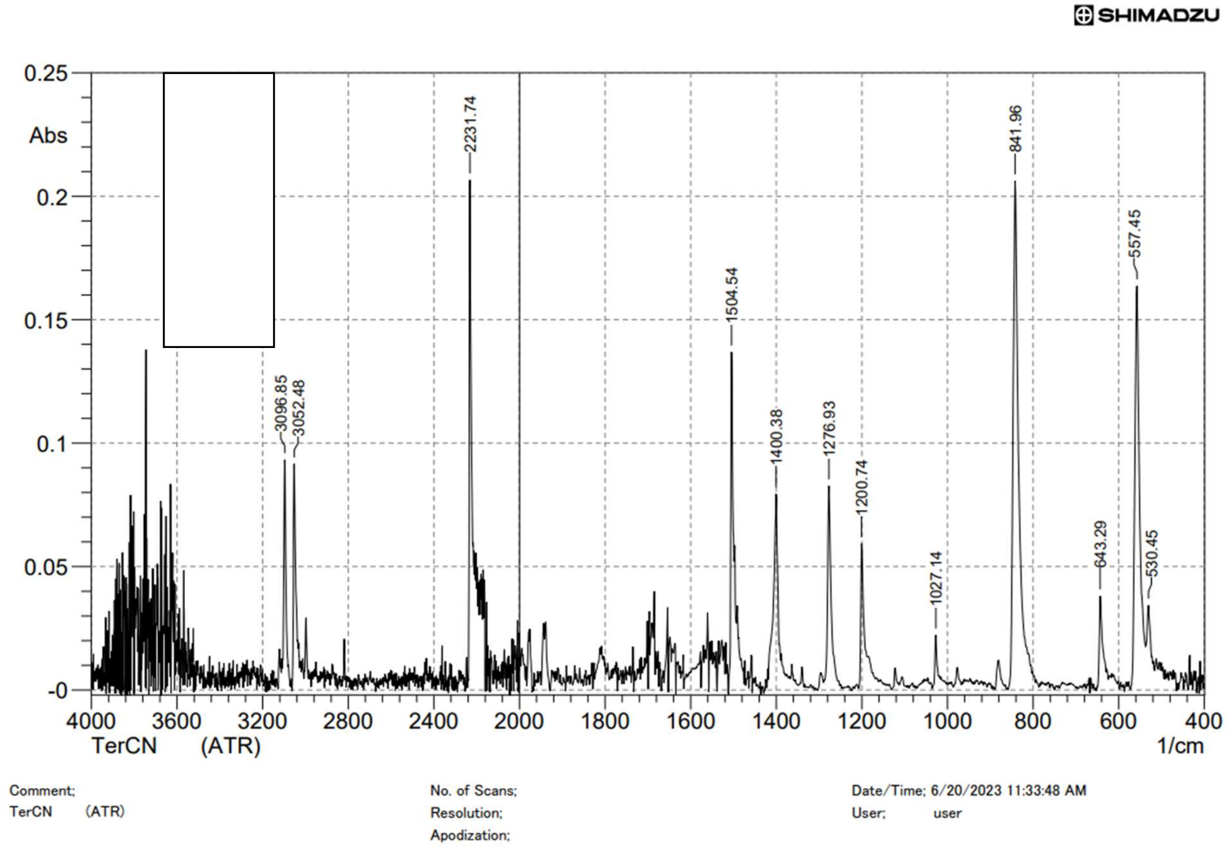

Figure S5. FTIR-ATR spectrum of **3**.

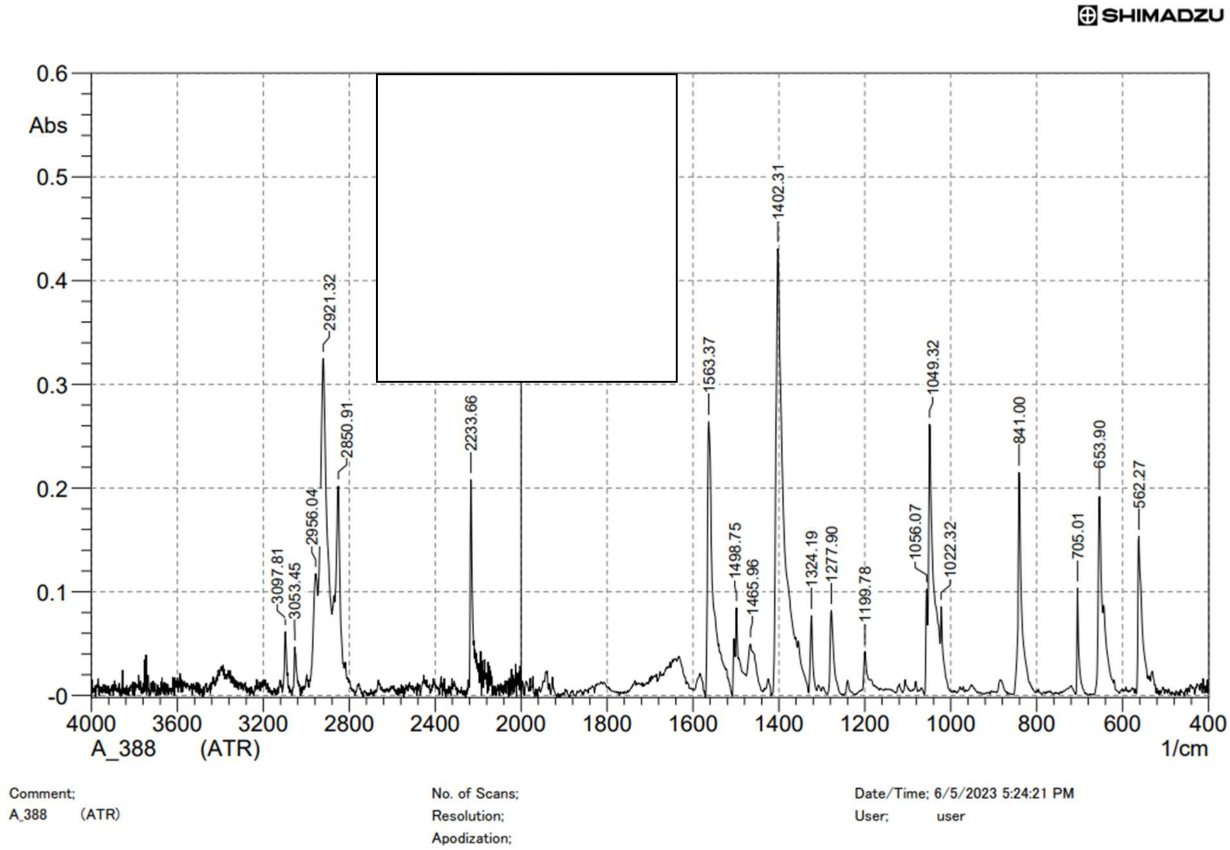

Figure S6. FTIR-ATR spectrum of **3**·2(1,3,5-FIB).

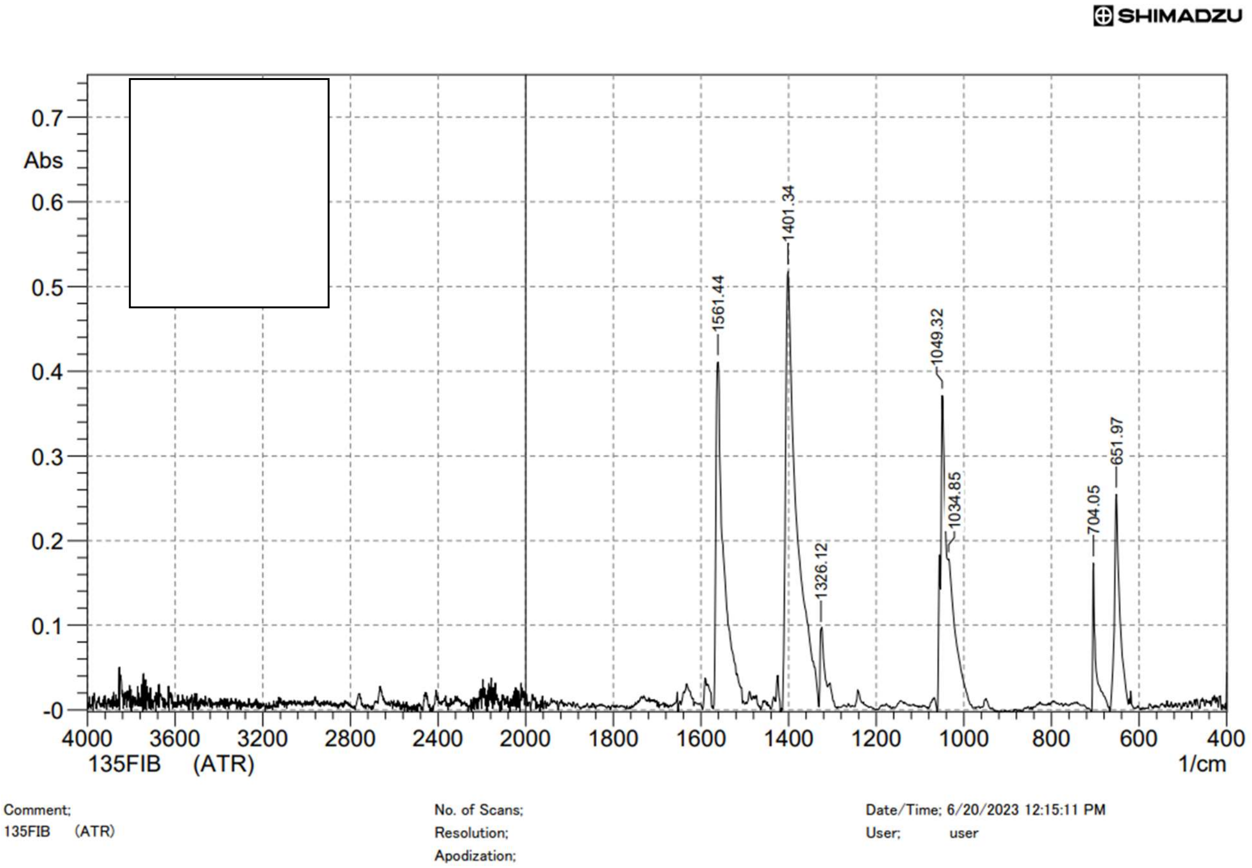

Figure S7. FTIR-ATR spectrum of 1,3,5-FIB.

PDXRD data

In all cases, experimental powder X-ray diffractograms are in an agreement with calculated patterns based on single-crystal XRD data. Side-peaks are from admixtures of the starting material(s).

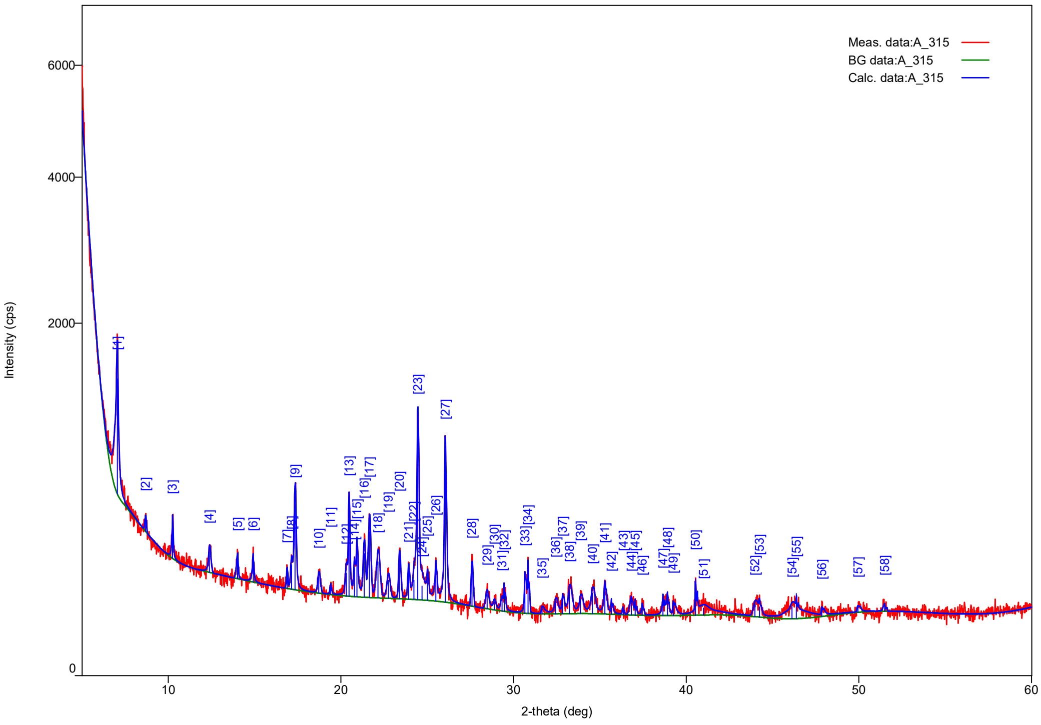

Figure S8. PDXRD of **1**·(1,3,5-FIB): experimental pattern (red) and calculated pattern (blue).

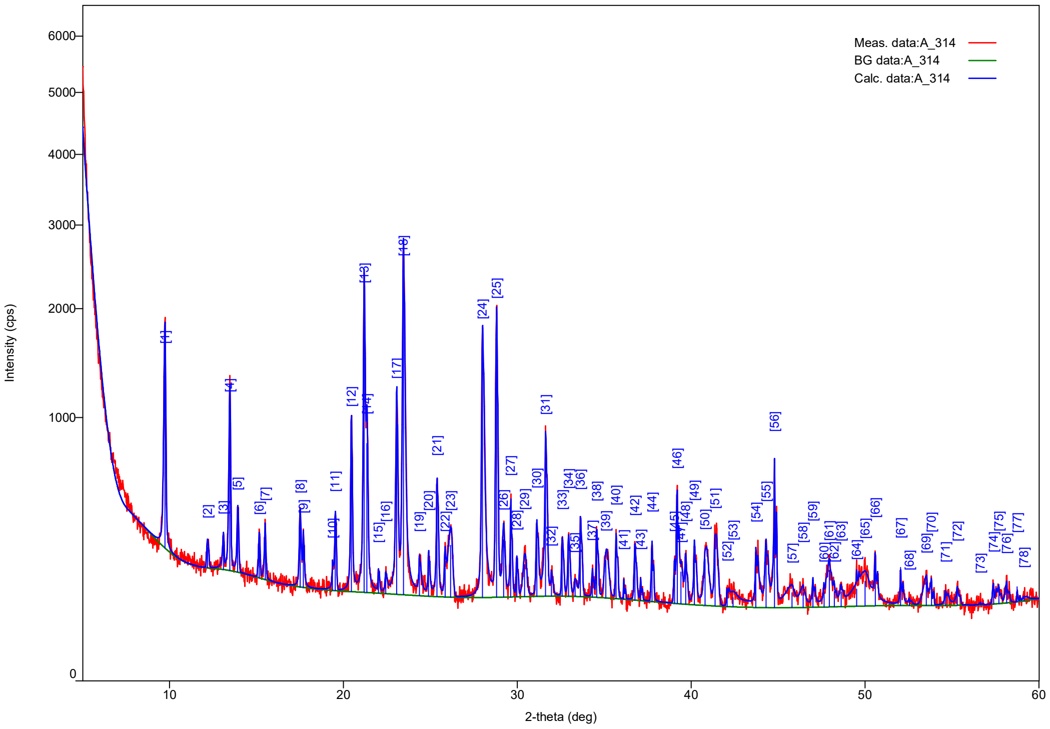

Figure S9. PDXRD of **2**·2(1,3,5-FIB): experimental pattern (red) and calculated pattern (blue).

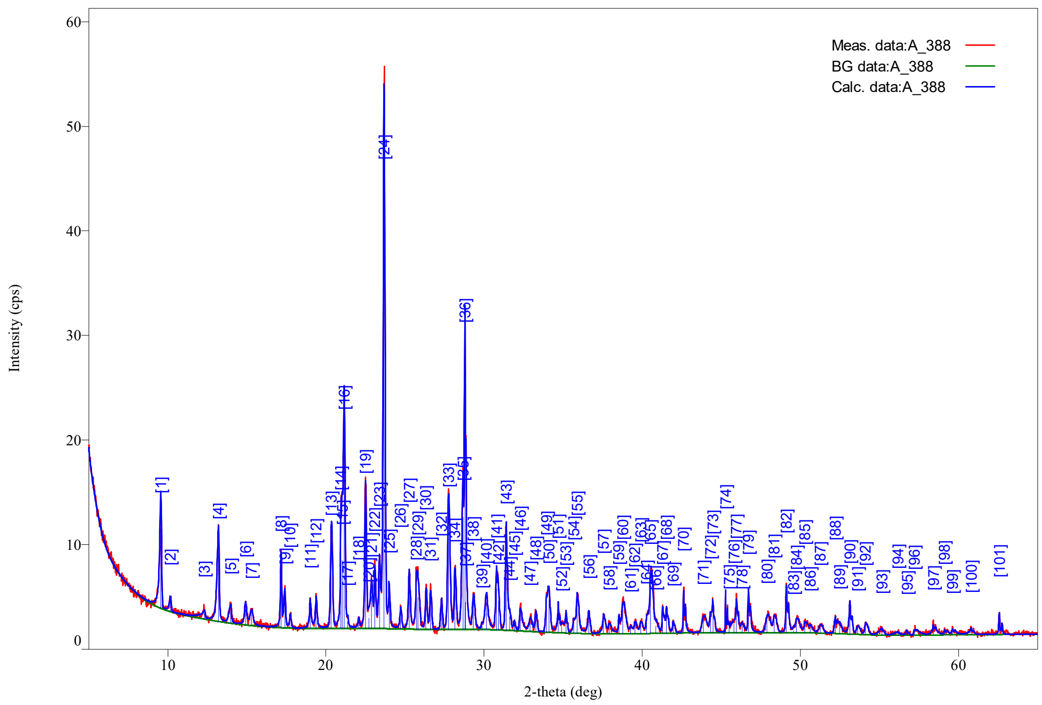

Figure S10. PDXRD of **3**·2(1,3,5-FIB): experimental pattern (red) and calculated pattern (blue).

Table S5. TG data.

Compound	Decomposition (beginning), °C	Decomposition (end point), °C	Total mass loss, %
1,3,5-FIB	258	299	98
**1**	193	232	99
**1**·(1,3,5-FIB)	202	241	98
**2**	146<br>196	168<br>218	24<br>45
**2**·2(1,3,5-FIB)	202	243	88
**3**	221	256	100
**3**·2(1,3,5-FIB)	192	245	100

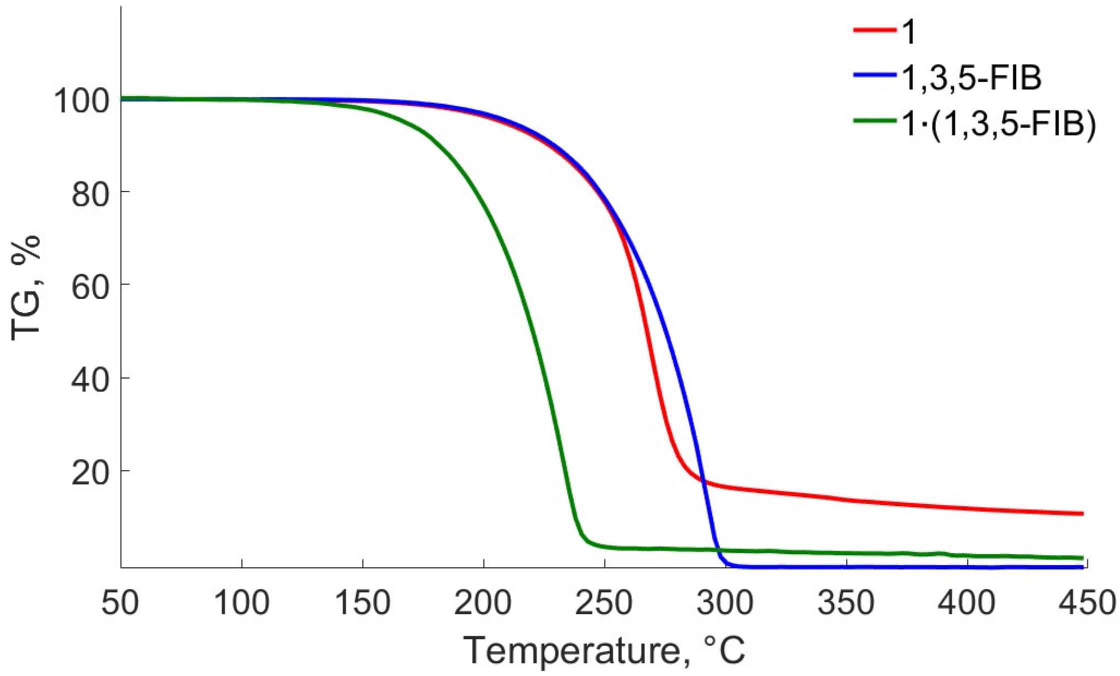

Figure S11. TG curve of **1**·(1,3,5-FIB).

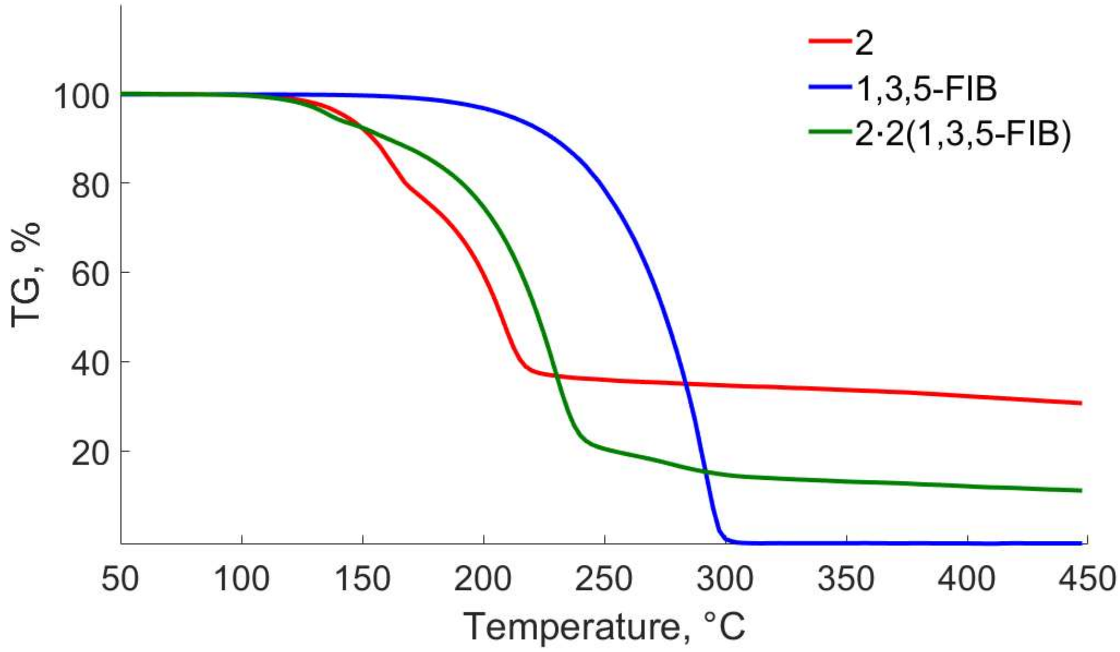

Figure S12. TG curve of **2**·2(1,3,5-FIB).

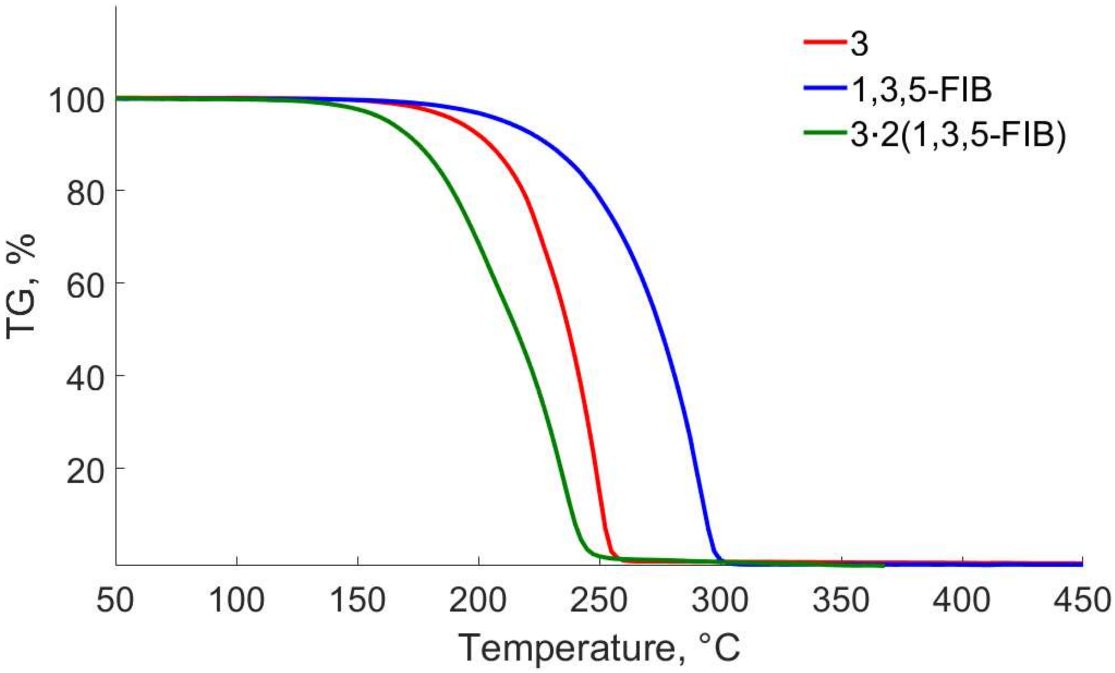

Figure S13. TG curve of **3**·2(1,3,5-FIB).

Cartesian coordinates for the studied cocrystals

Cartesian coordinates for optimized geometries of cocrystals:

Cartesian coordinate for [**1**·1,3,5-FIB] (in Å)

N -0.055647848 -0.117257599 -1.157201605

C 0.000000000 0.000000000 0.000000000

C -0.122683005 -0.265757307 -2.529516069

C 1.050394120 -0.583219648 -3.219590920

C -1.359799771 -0.091945646 -3.155982477

C 2.354347096 -0.756553540 -2.511122977

H 2.237756290 -0.732803407 -1.430689155

H 2.820690451 -1.705474567 -2.784646791

H 3.055097671 0.035369131 -2.791306103

C -2.590419023 0.249364418 -2.380775458

H -2.415385730 0.229370907 -1.308151229

H -2.949280508 1.248353805 -2.644997359

H -3.398117990 -0.449610617 -2.608900032

N -0.324717028 -0.729373365 -6.598999920

C -0.385741382 -0.874731583 -7.756289176

C -0.255081896 -0.565757835 -5.230328211

C -1.426598788 -0.243344411 -4.539468987

C 0.982466147 -0.734670967 -4.602999675

C -2.728900173 -0.068742329 -5.250866268

H -2.604967518 -0.084295419 -6.330676507

H -3.199749562 0.876478712 -4.972403952

H -3.428290173 -0.865558291 -4.981009954

C 2.211745073 -1.075243132 -5.380826276

H 2.033283914 -1.045179194 -6.452677894

H 2.566026045 -2.078513131 -5.126427001

H 3.022951966 -0.381969634 -5.147931475

I 2.641616740 1.239379498 8.356556833

I -2.752402110 -1.430889274 8.251247178

I 0.000000000 0.000000000 3.067833727

F -0.069798549 -0.106835416 9.267816565

F -2.111324307 -1.086712310 5.180149909

F 2.073317740 0.990805241 5.261996499

C 1.039343010 0.459108546 7.286863682

C -0.052633351 -0.087513155 7.942508076

C -1.127360367 -0.615020155 7.244292372

C -1.089288931 -0.583033847 5.858100875

C -0.020224710 -0.042765184 5.160416245

C 1.033889564 0.470751766 5.899739329

Cartesian coordinate for [**2**·1,3,5-FIB] (in Å)

I -1.454451816 -2.431001519 8.374243584

I 0.000000000 0.000000000 3.038236502

I 1.542009917 2.785956890 8.165654077

F -1.147170827 -1.954113370 5.267926786

F 1.177893791 2.095429055 5.106632230

F 0.053300759 0.212084308 9.230610570

C -0.567923722 -0.907082949 7.273254865

C -0.568609306 -0.934302780 5.886665653

C 0.014937848 0.068348752 5.128329522

C 0.611480920 1.120920873 5.804614801

C 0.636365479 1.190027322 7.189201941

C 0.040511030 0.165026139 7.906757077

N -0.050977036 0.080496465 -1.160822174

C 0.000000000 0.000000000 0.000000000

C -0.110344891 0.184130831 -2.532553360

C -0.177784510 1.442221738 -3.120614885

H -0.183550421 2.325243063 -2.495413805

C -0.101083068 -0.969334524 -3.308591121

H -0.048032943 -1.937290958 -2.828092225

N -0.284477901 0.499545593 -6.643382112

C -0.335210073 0.589122877 -7.807507446

C -0.226796690 0.394368251 -5.273406690

C -0.159486183 -0.863324974 -4.683062678

H -0.153447665 -1.746274412 -5.308237240

C -0.236205761 1.547152936 -4.495028357

H -0.289125297 2.515022985 -4.975554622

Cartesian coordinate for [**3**·1,3,5-FIB] (in Å)

I -1.372422403 -2.509853707 -5.312672805

I 0.000000000 0.000000000 0.000000000

I 1.616326230 2.712795628 -5.136934177

F -1.111190275 -1.987141493 -2.210058363

F 1.208961028 2.066219405 -2.074151497

F 0.145401354 0.121938825 -6.185665141

C -0.503530699 -0.969091092 -4.221680998

C -0.524685896 -0.976400224 -2.835024783

C 0.047332608 0.038355721 -2.083148608

C 0.653382129 1.081853549 -2.765883223

C 0.698068758 1.130424875 -4.150849790

C 0.113356012 0.094348211 -4.861648382

C 0.093008405 -0.005621989 4.189321250

N 0.000000000 0.000000000 3.043322070

C 0.213363524 -0.009341855 5.610721832

C 1.333793366 0.566620774 6.208390478

H 2.103787650 1.011070705 5.591334737

C -0.789199083 -0.586864038 6.388777347

H -1.653054579 -1.030200873 5.910883626

C 0.570418979 -0.012410623 9.784801373

N 0.669677333 -0.013966131 10.931468369

C 0.448760315 -0.011775691 8.362361131

C -0.671099842 -0.587795980 7.763433830

H -1.441102538 -1.031998364 8.380535901

C 1.450953246 0.565119795 7.583024680

H 2.314621225 1.008436975 8.061118570

Cartesian coordinate for **1** (in Å)

N -0.05449008526093 -0.10869448863794 -1.15871984147687

C 0.00220710984395 0.01841739647276 0.00067178781022

C -0.12259736564946 -0.26569298055287 -2.52827870429736

C 1.04890817852736 -0.58486925532665 -3.22087828410125

C -1.35925713851201 -0.09504352388113 -3.15726136569574

C 2.35179994735429 -0.76168124509719 -2.51057750057064

H 2.23148639351212 -0.72529621928036 -1.43084333034183

H 2.81042606926018 -1.71764244712838 -2.77334983493765

H 3.06007716616147 0.02096434629647 -2.79772998453194

C -2.59006983279207 0.24286244286063 -2.37995779254224

H -2.40973648900195 0.22141048672030 -1.30822391356193

H -2.95278890280551 1.24123368687655 -2.64123373386525

H -3.39674065434189 -0.45793545776321 -2.60692359480550

N -0.32289524465711 -0.72116816534487 -6.60266973794359

C -0.38222333636010 -0.86083813776890 -7.76056228512237

C -0.25473456467222 -0.56405527974200 -5.23276575420067

C -1.42632017035917 -0.24483567514821 -4.54076747400807

C 0.98196253465406 -0.73467010072360 -4.60438706446088

C -2.72941613565449 -0.06917050300271 -5.25060842304850

H -2.60975037327930 -0.10174993086319 -6.33055046993835

H -3.19083900427556 0.88454160464222 -4.98466256164731

H -3.43525350780391 -0.85487893583020 -4.96564601126630

C 2.21253847410522 -1.07364561653248 -5.38126703090838

H 2.03419467334078 -1.04892140778552 -6.45327623513938

H 2.57190296906306 -2.07380140902665 -5.12201988492455

H 3.02098766760319 -0.37590383343286 -5.15129644547363

Cartesian coordinate for **2** (in Å)

N -0.05146041776827 0.08012932369303 -1.15943977543787

C -0.00108972792290 -0.00367346417999 0.00503672266281

C -0.10984482697153 0.18477547436136 -2.53007763270237

C -0.17730621285050 1.44191663217096 -3.12128146005019

H -0.18325379199988 2.32528645310542 -2.49661392380521

C -0.10066976587513 -0.96758054432090 -3.30868406029317

H -0.04762222101921 -1.93546967424938 -2.82808833470787

N -0.28514286162811 0.49932478142264 -6.64524503027823

C -0.33644706968180 0.58851476770071 -7.80914738890588

C -0.22666296202503 0.39454425538208 -5.27463871197299

C -0.15916956205238 -0.86247944190559 -4.68336039219049

H -0.15321681303759 -1.74582021651103 -5.30810188243022

C -0.23579932672796 1.54689268850723 -4.49593183716285

H -0.28883744043971 2.51471896482345 -4.97669229272548

Cartesian coordinate for **3** (in Å)

C 9.69431253053338 -2.16186168090228 -0.12619261895163

N 8.67792336524782 -1.62975192126514 -0.03150851254158

C 10.95644543532329 -2.81851879980932 -0.24530416832973

C 11.99661251031167 -2.19741526753272 -0.93585501314831

H 11.84032578291058 -1.22170306718711 -1.37705647262141

C 11.14071217165521 -4.07556608947705 0.32924949655954

H 10.32531622934737 -4.54658180715989 0.86252535922099

C 14.66346412998652 -4.74426527595807 -0.59673108789232

N 15.68102634317069 -5.27332118512801 -0.69477998154395

C 13.40109042202318 -4.08845449171541 -0.47688335915507

C 12.36089146511748 -4.70933491010943 0.21361277818978

H 12.51717680765192 -5.68510111709004 0.65478780768350

C 13.21669597013829 -2.83133145678352 -1.05138050366816

H 14.03221412684040 -2.36048079572290 -1.58468127245063

Cartesian coordinate for 1,3,5-FIB (in Å)

I 2.63983497050123 1.24418016519156 8.35563967397901

I -2.75134133036216 -1.43522794191665 8.24956784098940

I 0.00440548496564 -0.01012854372766 3.08909428380451

F -0.06624597277009 -0.11175713294623 9.26154036739834

F -2.11593111019071 -1.07587492021489 5.17537254509716

F 2.07245284404238 0.99150558859650 5.25722589123339

C 1.04079261256524 0.46072282862595 7.28536165263353

C -0.05168306988867 -0.08920357428625 7.93828675215677

C -1.12930306424268 -0.61587277025464 7.24280715548217

C -1.09493433063397 -0.58137481915991 5.85728703898406

C -0.02041587352020 -0.04273318177737 5.16627511071365

C 1.03750356853400 0.47304001186958 5.89906297752800
