# Supplementary material for: Halogen Bonding Involving Isomeric Isocyanide/Nitrile Groups
Source: Int J Mol Sci. 2023 Aug 28;24(17):13324. doi: 10.3390/ijms241713324 (PMC10487382; doi:10.3390/ijms241713324)

checkCIF/PLATON report

You have not supplied any structure factors. As a result the full set of tests cannot be run.

THIS REPORT IS FOR GUIDANCE ONLY. IF USED AS PART OF A REVIEW PROCEDURE FOR PUBLICATION, IT SHOULD NOT REPLACE THE EXPERTISE OF AN EXPERIENCED CRYSTALLOGRAPHIC REFEREE.

No syntax errors found. CIF dictionary Interpreting this report

Datablock: 1ver0-22059_a_118

```
Bond precision:   C-C = 0.0063 A          Wavelength=1.54184

Cell:             a=13.2428(2)     b=17.1531(3)        c=9.4351(2)
                  alpha=90         beta=107.514(2)     gamma=90
Temperature:      100 K

                 Calculated                  Reported
Volume           2043.88(7)                  2043.88(7)
Space group      P 21/c                      P 1 21/c 1
Hall group       -P 2ybc                     -P 2ybc
Moiety formula   C12 H12 N2, C6 F3 I3        C6 F3 I3, 2(C6 H6 N)
Sum formula      C18 H12 F3 I3 N2            C18 H12 F3 I3 N2
Mr               694.00                      694.00
Dx,g cm-3        2.255                       2.255
Z                4                           4
Mu (mm-1)        36.316                      36.316
F000             1280.0                      1280.0
F000'            1281.13
h,k,lmax         16,20,11                    16,20,11
Nref             3823                        3818
Tmin,Tmax        0.090,0.336                 0.101,1.000
Tmin'            0.007

Correction method= # Reported T Limits: Tmin=0.101 Tmax=1.000
AbsCorr = MULTI-SCAN

Data completeness= 0.999           Theta(max)= 69.168

R(reflections)= 0.0369( 3614)                      wR2(reflections)=
                                                   0.0965( 3818)

S = 1.052                    Npar= 239
```

The following ALERTS were generated. Each ALERT has the format
test-name_ALERT_alert-type_alert-level.
Click on the hyperlinks for more details of the test.

Alert level B

```
PLAT315_ALERT_2_B Singly Bonded Carbon Detected (H-atoms Missing).         C1 Check
```

Author Response: C1 and C7 are isocyanide carbons

```
PLAT315_ALERT_2_B Singly Bonded Carbon Detected (H-atoms Missing).         C7 Check
```

Author Response: C1 and C7 are isocyanide carbons

Alert level C

```
ABSTY02_ALERT_1_C  An _exptl_absorpt_correction_type has been given without
            a literature citation. This should be contained in the
            _exptl_absorpt_process_details field.
            Absorption correction given as multi-scan
PLAT042_ALERT_1_C Calc. and Reported MoietyFormula Strings  Differ     Please Check
```

Alert level G

```
PLAT432_ALERT_2_G Short Inter X...Y Contact  I2       ..C1       .       3.08 Ang.
                                          -1+x,3/2-y,-3/2+z  =      4_474 Check
PLAT432_ALERT_2_G Short Inter X...Y Contact  I3       ..C7       .       3.04 Ang.
                                            1-x,1/2+y,1/2-z  =      2_655 Check
PLAT432_ALERT_2_G Short Inter X...Y Contact  F1       ..C12      .       2.93 Ang.
                                                      x,y,z  =      1_555 Check
PLAT434_ALERT_2_G Short Inter HL..HL Contact I1       ..F2       .       2.99 Ang.
                                           1-x,-1/2+y,1/2-z  =      2_645 Check
PLAT790_ALERT_4_G Centre of Gravity not Within Unit Cell: Resd.  #          2 Note
              C12 H12 N2
PLAT941_ALERT_3_G Average HKL Measurement Multiplicity ..........        2.8 Low
```

0 **ALERT level A** = Most likely a serious problem - resolve or explain
2 **ALERT level B** = A potentially serious problem, consider carefully
2 **ALERT level C** = Check. Ensure it is not caused by an omission or oversight
6 **ALERT level G** = General information/check it is not something unexpected

2 ALERT type 1 CIF construction/syntax error, inconsistent or missing data
6 ALERT type 2 Indicator that the structure model may be wrong or deficient
1 ALERT type 3 Indicator that the structure quality may be low
1 ALERT type 4 Improvement, methodology, query or suggestion
0 ALERT type 5 Informative message, check

Validation response form

Please find below a validation response form (VRF) that can be filled in and pasted into your CIF.

```
# start Validation Reply Form
_vrf_ABSTY02_1ver0-22059_a_118
;
PROBLEM: An _exptl_absorpt_correction_type has been given without
RESPONSE: ...
;
_vrf_PLAT042_1ver0-22059_a_118
;
PROBLEM: Calc. and Reported MoietyFormula Strings  Differ     Please Check
RESPONSE: ...
;
# end Validation Reply Form
```

It is advisable to attempt to resolve as many as possible of the alerts in all categories. Often the minor alerts point to easily fixed oversights, errors and omissions in your CIF or refinement strategy, so attention to these fine details can be worthwhile. In order to resolve some of the more serious problems it may be necessary to carry out additional measurements or structure refinements. However, the purpose of your study may justify the reported deviations and the more serious of these should normally be commented upon in the discussion or experimental section of a paper or in the "special_details" fields of the CIF. checkCIF was carefully designed to identify outliers and unusual parameters, but every test has its limitations and alerts that are not important in a particular case may appear. Conversely, the absence of alerts does not guarantee there are no aspects of the results needing attention. It is up to the individual to critically assess their own results and, if necessary, seek expert advice.

Publication of your CIF in IUCr journals

A basic structural check has been run on your CIF. These basic checks will be run on all CIFs submitted for publication in IUCr journals (*Acta Crystallographica*, *Journal of Applied Crystallography*, *Journal of Synchrotron Radiation*); however, if you intend to submit to *Acta Crystallographica Section C* or *E* or *IUCrData*, you should make sure that full publication checks are run on the final version of your CIF prior to submission.

Publication of your CIF in other journals

Please refer to the *Notes for Authors* of the relevant journal for any special instructions relating to CIF submission.

PLATON version of 06/07/2023; check.def file version of 30/06/2023

Datablock 1ver0-22059_a_118 - ellipsoid plot

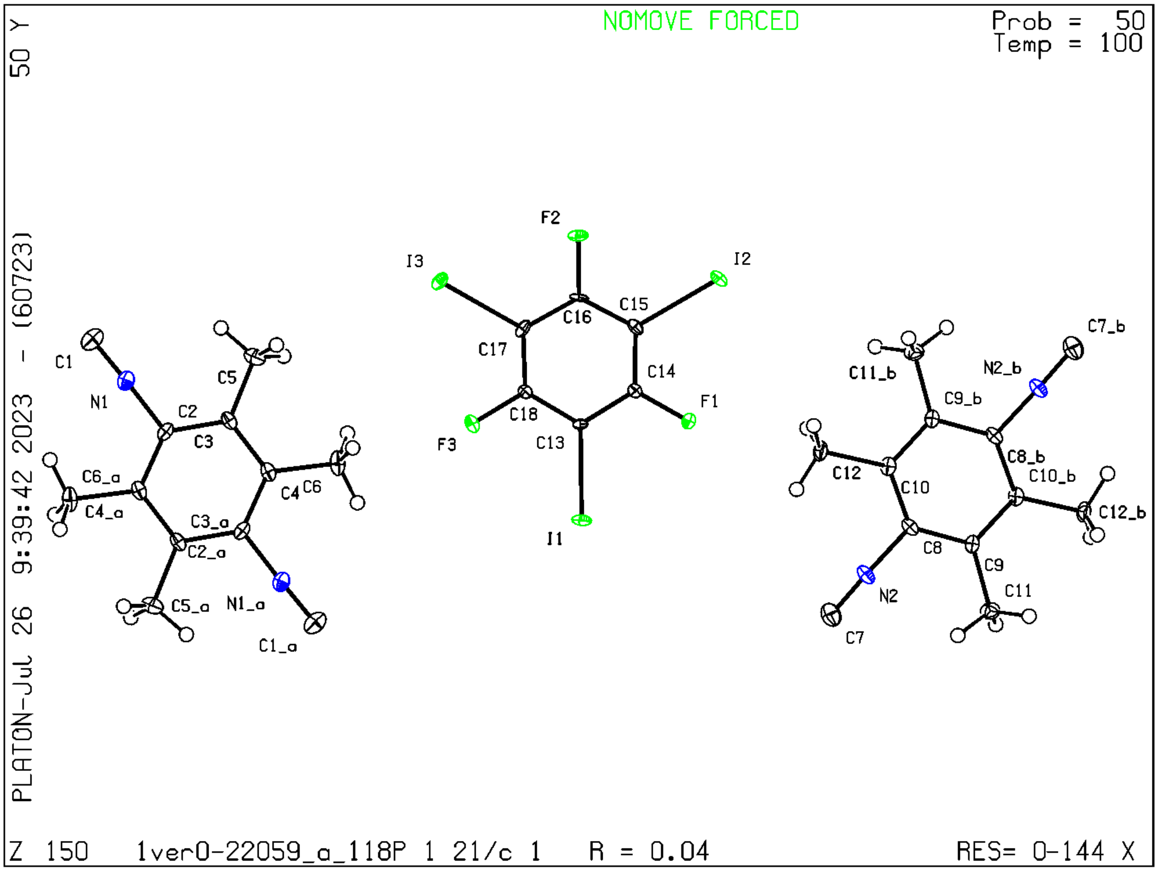

Supplement: Supplementary file 1 [file checkcif_a118.pdf › checkcif_a118.pdf]
